# The effect of learning management system on ICU nurses' sustained learning about safe blood transfusion: A quasi‐experimental study

**DOI:** 10.1002/hsr2.629

**Published:** 2022-06-06

**Authors:** Mojgan Falaki, Mehdi Ahmadinejad, Farideh Razban, Mohammad A. Najafipour, Neda Asadi

**Affiliations:** ^1^ Nursing Research Center Kerman University of Medical Sciences Kerman Iran; ^2^ Department of Anesthesiology, Fellowship of Critical Care, Faculty of Medicine, Shahid Bahonar Hospital Kerman University of Medical Sciences Kerman Iran; ^3^ Department of General Education, Afzalipour Faculty of Medicine Kerman University of Medical Sciences Kerman Iran

**Keywords:** blood transfusion, intensive care units, knowledge, LMS

## Abstract

**Background and Aims:**

Transfusion of blood products is an important part of the health care system. Since one of the significant challenges in nursing education is using an effective method that provides depth and stability of learning, this study aimed to assess using a learning management system (LMS) for intensive care unit (ICU) nurses' sustained learning about safe blood transfusion in southeast Iran.

**Methods:**

This was a quasi‐experimental study in southeast of Iran in 2021 in two groups, control and intervention. Two ICUs received lecture training and two ICUs received LMS. The samples 80 nurses were selected by random convenience sampling. In the LMS group, the educational content was presented using Edmodo software. The control group received no intervention except for traditional education (lecture). The questionnaires were completed immediately, 1 month, and 3 months after the intervention.

**Results:**

The mean score of knowledge immediately, 1 month and 3 months after the intervention were 9.53 ± 1.82, 9.46 ± 1.85, and 8 ± 2.94, in the lecture group and 8.91 ± 1.59, 9.47 ± 2.46, and 8.09 ± 1.94 in the LMS group, respectively. The mean score of practice immediately, 1 and 3 months after the intervention were 59.69 ± 39.6, 70.63 ± 7.4, and 83.70 ± 43.6 in the lecture group and 45.68 ± 55.5, 67.69 ± 4.56, and 35.70 ± 46.4 in the LMS group, respectively. The mean score of knowledge and practice in the two groups significantly increased immediately and 1 month and 3 months after intervention (*p* < 0.001). No significant difference was observed between the two groups (*p* > 0.05).

**Conclusions:**

LMS method has a significant effect on improving the sustained learning of ICU nurses, and no significant difference was observed between the two educational methods. Hence, considering the busy work schedule of nurses, lack of staff, and the impossibility of physical attendance in lecture classes, it seems that LMS‐based methods are appropriate alternatives to traditional learning methods.

## 1 INTRODUCTION

Blood transfusion (BT) is one of the most common procedures practiced in a hospital.^1^ The large number of transfusions performed in the hospital is related to intensive care units (ICUs),^2^ with more than a quarter of ICU patients undergoing transfusions of various blood products during their hospitalization.^3^ In spite of the clinical benefits of BT, there is the possibility of error at any stage of the transfusion process, which can cause serious and dangerous complications in the patient.^1^, ^4^ Nurses play a major role in this process, and more than half of the chain of BT depends on nursing practice.^5^ For this reason, BT requires sufficient knowledge and skill on the nurses' part.^6^ Providing continuous learning for nurses can improve their knowledge and skills and make desirable changes in the health system.^7^ Lecture‐based teaching is a method that has a long history in educational systems.^8^ For several reasons, such as low cost, the possibility of transferring a great volume of information to a large group in the shortest time, and also the proficiency of educators in using this method compared to other methods, lectures are the most common means of teaching in Iran.^9^, ^10^ However, in the current century, many educational activities are performed via the Internet and computer communications. E‐learning provides the conditions to simultaneously use three traditional learning methods: visual, auditory, and textual.^5^, ^11^

With the significant increase in the use of e‐learning, a variety of educational software has been developed in the form of learning management systems (LMSs) to facilitate online learning.^11^ LMSs help organize educational content and facilitate interaction between the learner and the instructor by creating private virtual classrooms. LMSs mainly allow doing tasks, handing in assignments, taking quizzes, asking questions, and receiving feedback from the instructor.^13^ Despite the widespread use of LMSs by learners, there is still no consensus on the effect of using these systems on learning in nursing. A literature review indicated that several studies had investigated the effect of using LMSs in teaching nursing students.^14^ The results of a study conducted by Saiz‐Manzanares et al.^14^ in Spain confirmed the positive effects of LMSs on teaching nursing students.^14^ Laili and Nashir reported a positive effect of using the combination of face‐to‐face and Edmodo‐based methods in nursing students in Indonesia.^15^ Howeber, Alhosban and Ismaile reported that the experience of nursing students in Saudi Arabia in using an LMS has often been negative, especially in communicating, interacting, and receiving feedback.^16^ Feng et al.^17^ conducted a meta‐analysis on 14 articles in which subgroup analyses showed that e‐learning programs effectively increased learners' knowledge and practice.^18^ But, Emami Sigaroudi et al. in Iran, concluded that the traditional education method is more desirable than the e‐learning method in terms of implementing the first principles of education.^19^ Researchers believe that any education leads to learning, but the depth and stability of learning using different methods are different.

Studies in the field of blood transfusion show a lack of knowledge and poor performance of nursing staff in this field. So far, little attention has been paid to the effects of virtual education on nurses' knowledge and practice in this regard. On the other hand, due to the busy professional of nursing, it is not economical to organize lecture classes and it is necessary to examine the effectiveness of virtual education. The most important issue in recent years is to evaluate the effectiveness of educational methods in sustained learning. Also, according to the researchers, educational software for nurses has not been used so far. And nurses have little knowledge of these soft wares. Due to the limited use of the Internet, the success of this educational method may be challenged. Hence, the present study was carried out to assess the effect of using learning management systems (LMSs) for ICU nurses' sustained learning about safe blood transfusion in southeast Iran.

## 2 METHODS

### 2.1 Sample and setting

The present quasi‐experimental study was conducted in four trauma ICUs of Shahid Bahonar Hospitals in Kerman, southeast of Iran in 2021. This governmental hospital has the highest rate of patient admission to intensive care units with various diagnoses. All 100 intensive care unit nurses were screened in terms of inclusion criteria. Inclusion criteria included having a bachelor's degree or higher in nursing and not having participated in BT educational programs within the 6 months leading to the study. By tossing coins two ICUs will receive random training, lecture training, and two ICUs will receive LMS. If both types of intervention are performed in one section, it is possible to transfer information. Therefore, it was decided that the nurses in each ward would receive only one type of intervention. Eighty ICU nurses were included in the study by convenience sampling method. Nurses were randomly divided into the two groups of control and LMS. Based on the study of Rafii et al.^11^ and also Type I error(*α*) = 0.05 and Type II error (*β*) = 0.02, The sample size in each group was estimated at 24 nurses. Considering the dropouts, the final sample size was determined 30 nurses per group (Figure S1).

### 2.2 Measures

The background information questionnaire included demographic information (such as age, gender, etc.) and information about BT (source of receiving information, experience of acute reactions, and type of reactions).

A self‐administered questionnaire, designed based on a literature review, was used to assess nurses' knowledge about the transfusion of blood products. The knowledge questionnaire consisted of 15 multiple‐choice questions (1 = correct answer and 0 = incorrect answer). The knowledge score was 0–15. A self‐administered questionnaire designed based on a literature review was used to assess nurses' practice in transfusion of blood products. The practice questionnaire consisted of 15 questions on a 5‐point Likert scale (1 = never to 5 = always). The range of scores was 15–75. The questionnaires were assigned to 10 faculty members of Razi School of Nursing and Midwifery, and they were modified based on the experts' opinions to evaluate the content validity of the questionnaires. The content validity index (CVI) of the knowledge questionnaire and the practice questionnaire were 0.92 and 0.96, respectively. The questionnaires were submitted to 20 people who had the same characteristics as the research sample to assess their reliability. Cronbach's alpha coefficients for the knowledge questionnaire and the practice questionnaire were 0.71 and 0.87, respectively.

### 2.3 Ethics approval and consent to participate

This study was approved by the Ethics Committee of the Kerman University of Medical Science with No. 98000432 and code of ethics No IR.KMU.REC.1398.305. All methods were carried out following relevant guidelines and regulations. Participation in this study was voluntary. All participants were informed about the study's objectives and process, and their informed consent was obtained. NA affirms that this manuscript is an honest, accurate, and transparent account of the study being reported; that no important aspects of the study have been omitted; and that any discrepancies from the study as planned (and, if relevant, registered) have been explained.

### 2.4 Intervention

Educational content was prepared using a literature review.^20^, ^21^ This content was provided to an ICU fellowship, 12 masters of ICU nursing with experience of working in the ICU, and five faculty members of the Nursing School, and their comments were considered and finally approved. This intervention was performed from December 2019 to April 2020. Both interventions started simultaneously to match the conditions of the two groups. In the lecture group, instruction was delivered using PowerPoint and whiteboard by an ICU fellowship and the physician in charge of the IRB BT Organization, in the conference hall of a university hospital in two 3‐h sessions (a total of 6 h) during two consecutive weeks. Before the intervention, an expert in educational technology held a technical instruction class for the LMS group to familiarize the participants with the platforms (registration, problem‐shooting, etc.). The learners were instructed to contact the researchers in case of any problems using the platform. In this group, the free Edmodo educational platform was used. This online platform was designed by O'Hara and Borg in 2008 and is available at www.Edmodo.com (20).

The educational content was presented on the Edmodo platform and was provided to the participants for 3 months. Educational content was presented in movies, posters, flashcards, and matching exercises. The educational videos were loaded onto the Edpuzzule online platform, and the flashcards were accessible on the Quizlet platform; links to these platforms were posted on Edmodo. A number of multiple‐choice questions, true and false questions and matching questions were prepared to practice the educational content in Edmodo software. The software provided immediate feedback on the correctness of the learners' answers. The software allowed learners to interact with each other in the chat room. In both the LMS and lecture groups, the learners completed the knowledge and practice questionnaires before the intervention and immediately, 1 month, and 3 months after the intervention. The two questionnaires were not provided to participants at the same time to prevent the impact of the knowledge questionnaire questions on learners' answers to the practice questionnaire questions. After each learner completed the knowledge questionnaire, this questionnaire was taken from the respondents, and then the practice questionnaire was handed out to them.

### 2.5 Statistical analysis

Data analysis was carried out using SPSS version 25. Descriptive statistics were used to analyze the participants' background data and the scores of each questionnaire. The Kolmogorov–Smirnov test indicated that the data had a normal distribution. The independent *t* test was used to compare the mean scores of knowledge and practice between the two groups, and *χ* ^2^ and Fisher's exact test was used to compare qualitative variables between the two groups. Analysis of variance (ANOVA) was used for intergroup and intragroup comparisons of mean scores at different times. *p* values less than 0.05 were considered statistically significant.

## 3 RESULTS

In the present study, 80 ICU nurses were assigned to two lecture and LMS learning methods. The mean age was 33.13 ± 5.39 years in the lecture group and 32.96 ± 6.99 years in the LMS group. There was no significant difference between the two groups concerning the demographic variables, and the two groups were homogenous in these variables (Table 1).

**Table 1:** Comparison of demographic variables in the study groups

Variable	Lecture	LMS	Statistical analysis	*p*‐value
	*N* (%)	*N* (%)		
Gender			Fisher exact test = 0.82	0.7
Female	29 (93.5)	45 (91.8)		
Male	2 (6.5)	4 (8.2)		
Education level			*χ* ^2^ = 0.33	0.7
Bachelor degree	29 (93.5)	44 (89.8)		
Master degree	2 (6.5)	5 (10.2)		
Position			Fisher exact test = 3	0.08
Nurse	31 (100)	46 (93.9)		
Supervisor	0 (0)	3 (6.1)		
Experience in BT			Fisher exact test = 95/1	0.16
Yes	29 (96.7)	49 (100)		
No	1 (3.3)	0 (0)		
How to get information about BT			*χ* ^2^ = 3.28	0.1
Personal	4 (12.9)	15 (30.6)		
Workplace	27 (87.1)	34 (69.4)		
Dealing with the acute reaction of BT			*χ* ^2^ = 0.52	0.49
Yes	12 (7.38)	23 (46.9)		
No	19 (3.61)	26 (53.1)		
Type of reactions			*χ* ^2^ = 1.67	0.64
Rash	0 (0)			
Hematuria	22 (71)	2 (4.1)		
Fever	8 (8.25)	32 (65.3)		
Tachycardia	1 (2.3)	12 (24.5)		

Abbreviations: BT, blood transfusion; LMS, learning management system.

The mean score of knowledge immediately, 1 month and 3 months after the intervention were 9.53 ± 1.82, 9.46 ± 1.85, and 8 ± 2.94, in the lecture group and 8.91 ± 1.59,9.47 ± 2.46, and 8.09 ± 1.94 in the LMS group, respectively. And the mean score of practice immediately, 1 month and 3 months after the intervention were 59.69 ± 39.6, 70.63 ± 7.4, and 83.70 ± 43.6 in the lecture group and 45.68 ± 55.5, 67.69 ± 4.56, and 35.70 ± 46.4 in the LMS group, respectively. The independent *t* test revealed no significant difference between the two groups' mean knowledge and practice scores before the intervention (*p* > 0.05). No significant difference was observed between the two groups in knowledge and practice immediately, 1 month, and 3 months after the intervention (*p* > 0.05). The result of one‐way ANOVA test showed that the knowledge and practice of nurses in 1 month and 3 months later had a significant incremental change in both groups compared to the results before the intervention (*p* < 0.001) (Table 2).

**Table 2:** Comparison of knowledge and practice in the study groups; before and immediately, 1 month, and 3 months after the intervention

Variable	Groups	Scores (mean ± *SD*)				Repeated measures	
		Before intervention	Immediately after intervention	1 month after intervention	3 months after intervention	*F* **	*p* value
Knowledge	Lecture	7.48 ± 2.09	9.53 ± 1.82	9.46 ± 1.85	8 ± 2.94	12	<0.001
	LMS	7.12 ± 1.47	8.91 ± 1.59	9.47 ± 2.46	8.09 ± 1.94	22.7	<0.001
	*p*‐value*	0.43	0.11	0.57	0.48		
Practice	Lecture	63.91 + 63.4	59.69 ± 39.6	70.63 ± 7.4	83.70 ± 43.6	12.47	<0.001
	LMS	65.74 ± 4.78	45.68 ± 55.5	67.69 ± 4.56	35.70 ± 46.4	44.08	<0.001
	*p*‐value*	0.09	0.41	0.37	0.98		

Abbreviations: ANOVA, analysis of variance; LMS, learning management system; *SD*, standard deviation.

* Significance level of <0.05.

** One‐way ANOVA.

**Figure 1:**
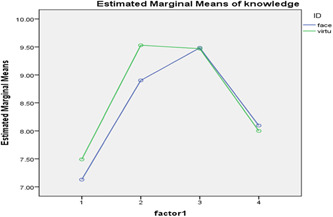
Repeated measures of the mean scores of knowledge in the two groups in the four measurements.

**Figure 2:**
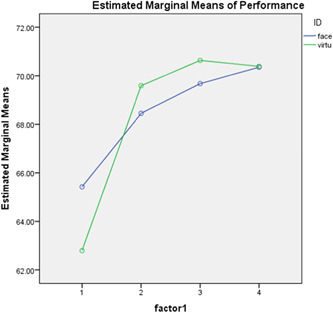
Repeated measures of the mean scores of performance in the two groups in the four measurements.

Figures 1 and 2, illustrate the repeated measures analysis of the mean scores of knowledge and practice in the two groups in the four measurements.

## 4 DISCUSSION

The present study aimed to compare the effects of lecture and LMS on the BT knowledge and practice of nurses in ICUs in southeastern Iran. The results showed that ICU nurses' mean scores of knowledge and practice in the two groups increased significantly compared to before the intervention. Also, no significant difference was observed between the two groups in this regard. Hence, based on the results, the two interventions effectively improved the knowledge and practice of ICU nurses. In the study conducted by Rafiei et al. to evaluate the effect of educational workshops and multimedia training on nurses' knowledge and practice in the field of BT on nurses working in hospitals, the results revealed that both educational methods enhanced the knowledge of the nurses and no significant difference was observed between the two methods in this regard.^11^ A quasi‐experimental study in Egypt on pediatric nurses' knowledge and practice in BT showed that nurses' knowledge and practice were significantly increased 3 months after the lecture.^22^ The results of the mentioned studies are consistent with those of the present study in terms of the effectiveness of lecture and multimedia training in nurses' knowledge about BT and 3 months after the intervention.

Elewa and Elkattan^23^ also showed that various training strategies, including lectures, positively affect nurses' practice in BT. The results suggested that educational programs positively impacted the nurses' practice in BT in thalassemia patients and improved the quality of nursing care, increasing patient satisfaction.^23^ The results of the study conducted by Hugenholtz et al.^23^ confirmed the results of the present study and showed that both e‐learning and lecture‐based training methods were effective in increasing learners' knowledge, and there was no statistically significant difference between the two groups.^24^ Feng et al.^17^ conducted a meta‐analysis on 14 articles in which subgroup analyses showed that e‐learning programs effectively increased learners' knowledge and practice.^18^ Farshi et al. showed that in the area of nursing care training on air rescue by two methods, lecture and electronic, the mean score of knowledge after intervention in the two groups showed a significant difference, and this increase in the lecture group was significantly higher.^25^ Saiz‐Manzanares et al.^14^ confirmed the positive effects of LMSs on teaching nursing students.^14^ Laili and Nashir reported a positive effect of using the combination of face‐to‐face and Edmodo‐based methods in nursing students.^15^

In contrast to the present study results, the study conducted by Moazami et al.^26^ on 35 dental students to compare virtual and traditional training of dental students showed that virtual training was more effective than traditional training.^26^ One of the reasons for the difference in results of these two studies is the different nature of educational content and the tools used. Also,  Hashemiparast et al. showed that the traditional education method is more desirable than the e‐learning method in terms of implementing the first principles of education in nursing students. The difference between the tools used and the research community can be the reasons for the difference.^27^ Hashemiparast et al.'s finding showed that in the area of awareness of administrative staff of clinical departments by two methods, lecture and electronic, the mean score of awareness of administrative staff was significantly higher in the lecture group.^27^ Perhaps the differences in instrument and aim of the study could be the causes of this contradiction.

## 5 LIMITATION

This study had its limitations. Lack of familiarity and knowledge of nursing care providers about the virtual method used. To solve this problem, a training session was held to get acquainted with the installation and use of the relevant software. Also, because the present study was conducted in a hospital and the learners of each intervention group may interact with each other, the learners of each intervention group were asked not to provide information to the other group during the study. To reduce the effect of information exchange, each type of intervention was performed in one section. Another limitation of this study was the reluctance of some nurses to participate in the study. With explaining the objectives of the study, we tried to attract their cooperation. It is recommended that similar studies be performed in multiple hospitals with larger sample sizes.

## 6 CONCLUSION

The present study results showed that both lecture and LMS methods have a significant effect on improving the level of knowledge and practice of ICU nurses, and no significant difference was observed between the two interventions. Considering the busy work schedule of nurses, lack of staff, and the impossibility of physical attendance in lecture classes, it seems that LMS‐based methods are appropriate alternatives to traditional learning methods.

## WHAT IS ALREADY KNOWN ABOUT THE TOPIC?

- Evidence‐based knowledge and skills are essential for critical care nurses to enhance the quality of blood transfusion procedures.
- Standardized education is required for sufficient knowledge and skills of nurses in blood transfusion.
- Providing continuous learning for critical care nurses can improve their knowledge and skills about the principles of blood transfusion.

## WHAT THIS PAPER ADDS?

- Using a learning management system (LMS) is an effective method for improving the level of knowledge and practice of ICU nurses about principles of blood transfusion, and it is as effective as the traditional method.
- Considering the busy work schedule of nurses, the lack of staff, and the impossibility of physical attendance in lecture classes, it seems that LMS‐based methods are appropriate alternatives to traditional learning methods.

## AUTHOR CONTRIBUTIONS

**Mojgan Falaki**: Conceptualization; data curation; investigation; software; writing—original draft. **Mehdi Ahmadinejad**: Conceptualization; supervision; writing—review & editing. **Farideh Razban**: Conceptualization; methodology; writing—original draft. **Mohammad Ali Najafipour**: Conceptualization; software. **Neda Asadi**: Conceptualization; funding acquisition; methodology; project administration; writing—original draft; writing—review & editing.

## CONFLICTS OF INTEREST

The authors declare no conflicts of interest.

## TRANSPARENCY STATEMENT

The lead author affirms that this manuscript is an honest, accurate, and transparent account of the study being reported, that no important aspects of the study have been omitted, and that any discrepancies from the study as planned have been explained.

## Supporting information

Supplementary information.

## Data Availability

The data are available upon request to the corresponding author after signing appropriate documents in line with ethical application and the decision of the Ethics Committee. Also, the authors confirm that the data supporting the findings of this study are available within the article.
