# Supplementary material for: The effect of learning management system on ICU nurses' sustained learning about safe blood transfusion: A quasi‐experimental study
Source: Health Sci Rep. 2022 Jun 6;5(4):e629. doi: 10.1002/hsr2.629 (PMC9169337; doi:10.1002/hsr2.629)
Supplement: Supplementary file 1 — Supplementary information. [file HSR2-5-e629-s001.doc]

Fig. S1: Flow diagram of the study according to CONsolidated Standards Of Reporting Trials (CONSORT).

**Allocation**

**Analysis**

**Follow-Up**

**Enrollment**

**Allocation**

**Analysis**

**Follow-Up**

**Enrollment**

Assessed for eligibility (n=100)

Excluded (n= 20)

♦  Not meeting inclusion criteria (n=15)

♦  Declined to participate (n= 5)

♦  Other reasons (n= 0)

Analysed (n=49)
 Excluded from analysis (give reasons) (n= 0 )

Lost to follow-up (give reasons) (n=0)

Discontinued intervention (give reasons) (n=0)

Allocated to intervention (LMS group) (n=49)

♦ Received allocated intervention (n=49)

♦ Did not receive allocated intervention (give reasons) (n= 0 )

Lost to follow-up (give reasons) (n=0)

Discontinued intervention (give reasons) (n=0)

Allocated to control (lecture group) (n=31)

♦ Received allocated intervention (n=31)

♦ Did not receive allocated intervention (give reasons) (n= 0)

Analysed (n=31)
♦ Excluded from analysis (give reasons) (n=0 )

Randomized (n=80)

Assessed for eligibility (n=100)

Excluded (n= 20)

♦  Not meeting inclusion criteria (n=15)

♦  Declined to participate (n= 5)

♦  Other reasons (n= 0)

Analysed (n=49)
 Excluded from analysis (give reasons) (n= 0 )

Lost to follow-up (give reasons) (n=0)

Discontinued intervention (give reasons) (n=0)

Allocated to intervention (LMS group) (n=49)

♦ Received allocated intervention (n=49)

♦ Did not receive allocated intervention (give reasons) (n= 0 )

Lost to follow-up (give reasons) (n=0)

Discontinued intervention (give reasons) (n=0)

Allocated to control (lecture group) (n=31)

♦ Received allocated intervention (n=31)

♦ Did not receive allocated intervention (give reasons) (n= 0)

Analysed (n=31)
♦ Excluded from analysis (give reasons) (n=0 )

Randomized (n=80)
